# A well‐tolerated new amino acid–based formula for cow's milk allergy

**DOI:** 10.1002/iid3.286

**Published:** 2020-02-28

**Authors:** Vincenzo Fierro, Rocco L. Valluzzi, Claudia Banzato, Ma A. Plaza, Montserrat Bosque, Marcel Íbero, Luis A. Z. Echeverría, Maurizio Mennini, Lamia Dahdah, Roser de Castellar, Gloria Tort, Jesus Jiménez

**Affiliations:** ^1^ Pediatric Allergy Unit Bambino Gesù Hospital Roma Italy; ^2^ Azienda Ospedaliera Universitaria Integrata Verona Italy; ^3^ Hospital Sant Joan de Deu Barcelona Spain; ^4^ Consorci Sanitari Parc Taulí Sabadell Spain; ^5^ Hospital de Terrassa Terrassa Spain; ^6^ Hospital Severo Ochoa Laganès Spain; ^7^ Laboratorios Ordesa Sant Boi de Llobregat Spain

**Keywords:** Allergy<Processes, amino acid formula, children, cow's milk allergy, toddler

## Abstract

**Objectives:**

Infants with cow's milk allergy (CMA) are in need of a substitute formula up to 2 years. The are three requisites for a substitute of milk in CMA: tolerability, nutritional adequacy, and cost‐effectiveness. We evaluate here the tolerability of a new amino acid–based infant formula for the management of CMA.

**Methods:**

In a phase III/IV prospective, multicentre, open‐label, international study, infants and children with immunoglobulin E‐mediated CMA were exposed to a diagnostic double‐blinded, placebo‐controlled food challenge with a new amino acid formula by Blemil Plus Elemental using Neocate as the placebo. If tolerant to it, the study formula was integrated into the patients’ usual daily diet for 7 days. Efficacy on day 7 was assessed in terms of symptoms associated with CMA, amount of formula consumed, nutritional and energy intake, and anthropometric data.

**Results:**

Thirty children (17 M and 13 F; median age, 1.58; range, 0.08‐12.83 years) completed the open challenge and were able to consume the study formula for at least 7 days. No signs or symptoms of allergic reactions were recorded among children assuming either the test or the control formula, with a lower 95% one‐sided confidence interval for the proportion of subjects who did not experience allergic reactions above 90%. Sixteen patient under the age of two continued with the optional extension phase.

**Conclusions:**

The study formula meets the American Academy of Pediatric criteria for hypoallergenicity and is well tolerated in short‐term use. During optional phase, growth of the patients was not hindered by the study formula.

## Graphical abstract

This study provides data on a new amino acid formula, documenting the hypoallergenicity of such a preparation in a cohort of children with milk allergy. These children are in need of a substitute formula especially up to 2 years. We designed a study in compliance with the rules and procedures indicated by the American Academy of Pediatric regarding studies on new special formulas to be included in the therapeutic options.

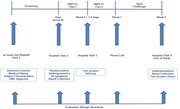

**Abbreviations**
AAF: amino acid formula
AAP: American Academy of Pediatrics
CMA: cow's milk allergy
CoMiSS: cows’ milk symptoms‐based clinical score
DBPCFC: double‐blinded, placebo‐controlled food challenge
eHF: extensively hydrolyzed formula
FAS: full analysis set
PP: per protocol

## 1 INTRODUCTION

**What's known:**

Tolerability of amino acid formula (AAF) is high, but occasional allergic reactions have been documented, due to accidental cross‐contamination with milk proteins when the same equipment is used for producing both AAF and extensively hydrolyzed formula. Previous data were published about hypoallergenicity of AAF as Neocate, Sineall, and Novalac.

**What's new:**

A new amino acid formula (Blemil Plus Elemental) is a safe product, meeting the formal documentation of allergenic safety. As stated by the American Academy of Pediatrics definition, it can be defined hypoallergenic.

Food allergys affect up to 6% of children.^1^ Cow's milk allergy (CMA) is the commonest food allergy in infancy, representing 2% to 3% of allergic disease in the developed world.^2^, ^3^, ^4^, ^5^ CMA peaks at 1 year, when milk is a staple food. Thus, although the majority of these infants achieve spontaneous tolerance after 2 to 3 years,^5^, ^6^ in case of unavailability of breastfeeding, they are in need of a substitute. There are three requisites for a substitute of milk in CMA: tolerability (it must not determine allergy symptoms), nutritional adequacy (it must allow normal growth), and cost‐effectiveness.^7^ This is particularly true in children with multiple food allergies,^8^ often associated with the most severe forms of CMA, including severe eczema^9^ and anaphylaxis.^10^ These are the infants for which an amino acid formula (AAF) is recommended over vegetable and extensively hydrolyzed formula (eHF), due to the risk of sensitization to soy or residual milk proteins.^8^, ^11^, ^12^ By definition, a hypoallergenic formula must clinically demonstrate tolerance in 90% of infants or children with confirmed CMA with 95% confidence.^13^ We sought to verify the hypoallergenicity of a new amino acid‐based formula in infants and children with documented CMA and the short‐term effects on growth.

## 2 METHODS

This is a prospective, multicentre, international study. The primary outcome was the incidence of immediate and/or delayed allergic reactions to a double‐blinded, placebo‐controlled food challenge (DBPCFC) and to a subsequent open challenge with Blemil Plus Elemental. Secondary endpoints were tolerability parameters of gastrointestinal, respiratory, and dermatological symptoms, and nutritional parameters, including growth and study product intake.

### 2.1 Patients

Children up to 12 years with immunoglobulin E (IgE)–mediated CMA were enrolled. CMA was confirmed when one of the following criteria was satisfied, within 6 months before study start (visit 2): positive DBPCFC with cow's milk or positive open or single‐blind oral food challenge with cow's milk, carried out under the supervision of a specialist in children with clear immediate reactions and a positive test for specific IgE (in serum [sIgE > 0.35 KUI/L] or skin prick test [SPT] ≥ 3 mm). Convincing allergic symptoms were reported following exposure to milk or milk‐containing food products and detectable serum milk–specific IgE or positive SPT. Children with chronic diseases, congenital anomalies, or major gastrointestinal disease/abnormalities were excluded. Existing illnesses that could interfere with formula acceptance or with the identification of allergic reactions at challenge prejudiced the inclusion in the study. Children with unstable asthma and severe uncontrolled eczema were included when clinically stabilized. Thus, children with life‐threatening anaphylactic reaction to milk were included only if in the least 2 years they were free from anaphylaxis.

Diagnosis was confirmed for 14 patients enrolled after a DBPCFC and for 12 patients after an open food challenge. Four patients had a clear clinical history of an anaphylactic reaction in the 6 months before the screening visit that was considered diagnostic^5^ (enrolled patients data, characteristic, family history, and comorbidity described on Tables 1, 2, 3). The study started when the patient ceased breastfeeding and had been free of clinical symptoms or with controlled stable symptoms for at least 1 week. Written informed consent from one or both parents (depending on the local legislation) or legal representative was obtained for the participation to the study and for each of the challenge procedures.

**Table 1:** Composition of the study formula

Per 100 kcal	Test formula (Blemil Plus Elemental)	Control formula (Neocate)
Protein source	Amino acids	Amino acids
Protein (g/100 kcal, % kcal)	2.6, 10.4%	3.1, 12%
Fat source	Vegetable oils (palm oil, palm kernel oil, rapeseed oil, sunflower oil, sunflower high oleic oil), and MCT oil (palm and/or coconut oil), DHA	Refined vegetable oil (sunflower high oleic, sunflower oil, soy oil) and MCT oil (palm and/or coconut oil), AA, DHA
Fat (g/100 kcal, % kcal)	5.2, 47%	4.5, 41%
Carbohydrate source	Corn starch, maltodextrin	Corn syrup solids
Carbohydrate (g/100 kcal, % kcal)	10.6, 42.6%	11.7, 47%
Nucleotides	Yes	No

**Table 2:** Patients characteristic

	ITT		PP	
	N = 39		N = 30	
Age of baseline, y				
Min	0.0		0.0	
Max	12.8		12.8	
Mean (SD)	2.1 (2.518)		2.36 (2.809)	
Age range at baseline, y	n (%)			
<2	25 (64.1)		18 (60.0)	
2‐5	12 (30.8)		10 (33.3)	
6‐12	1 (2.6)		1 (3.3)	
>12	1 (2.6)		1 (3.3)	
Gender	n (%)			
Male	19 (48.7)		17 (56.7)	
Female	20 (51.3)		13 (43.3)	
Race/ethnicity	n (%)			
Asian	1 (2.6)		…	
Black	1 (2.6)		1 (3.3)	
Caucasian	34 (87.2)		28 (93.3)	
Hispanic	1 (2.6)		1 (3.3)	
Not specified	2 (5.1)		…	

Abbreviations: ITT, intention‐to‐treat; PP, per protocol.

**Table 3:** Parents characteristic

Father (N = 28)		Mother (N = 30)	
Age, y		Age, y	
Min	28	Min	22
Max	52	Max	43
Mean (SD)	37.5 (5.9)	Mean (SD)	33.9 (5.18)
Race/ethnicity	n (%)	Race/ethnicity	n (%)
Asian	1 (2.56)	Asian	1 (2.56)
Black	1 (2.56)	Black	1 (2.56)
Caucasian	32 (82.05	Caucasian	33 (84.62)
Hispanic	1 (2.56)	Hispanic	1 (2.56)
Not Specified	4 (10.26)	Not Specified	3 (7.69)
Any allergy, n (%)	15 (38)	Any Allergy, n (%)	9 (23)
CMA, n (%)	0 (0)	CMA, n (%)	1 (3)
First son, n (%)		21 (54%)	

Abbreviation: CMA, cow's milk allergy.

### 2.2 Study design

**Figure 1:**
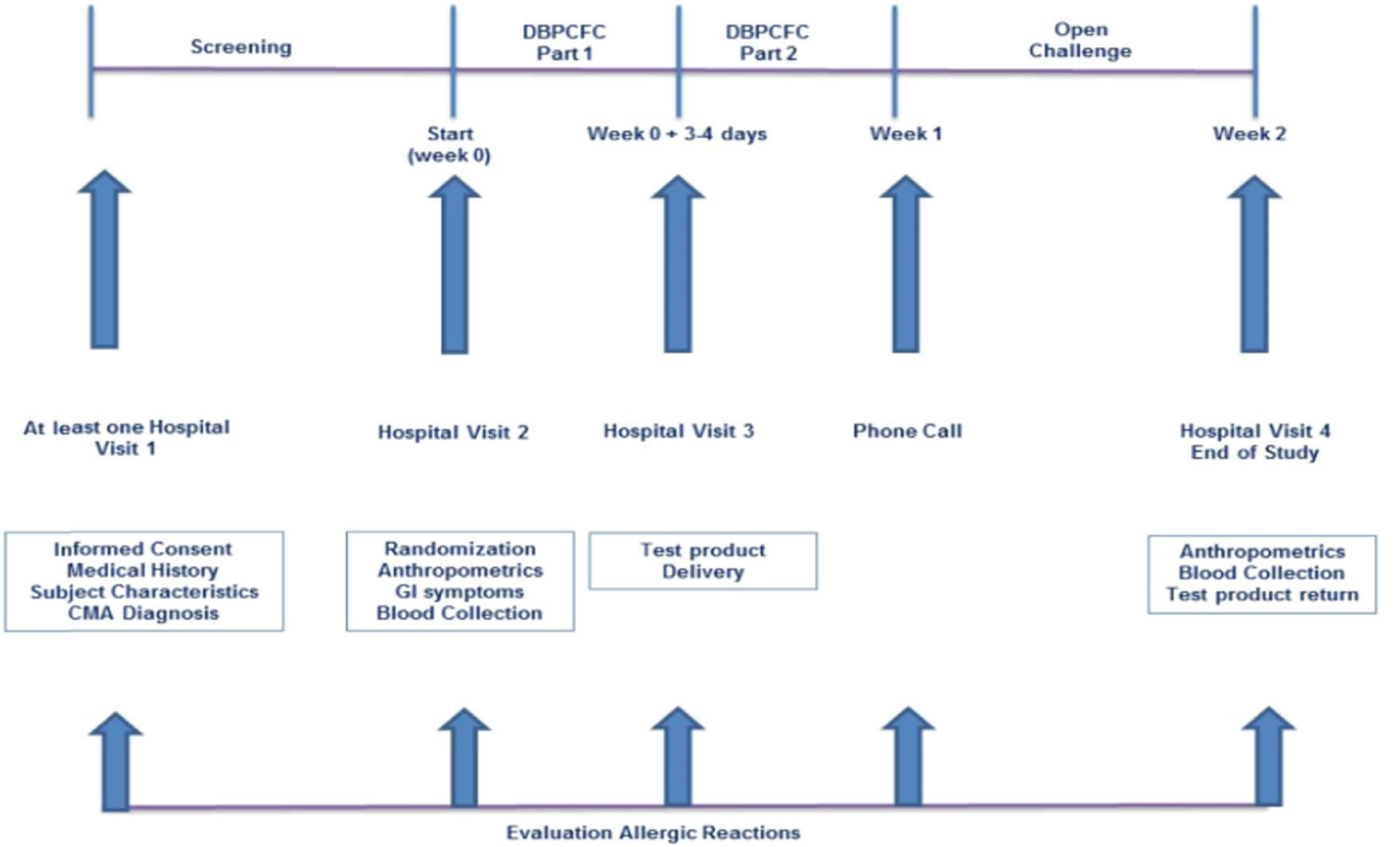
Flowchart of the study

The test product is a powdered amino acid‐based infant formula indicated for the management of CMA. Its composition (Table 1) satisfies the American and European nutritional standards.^14^, ^15^ The study formula tolerability was tested at DBPCFC and, when tolerated, it was included in the patients’ diet for at least 7 days. If the formula was tolerated during this period, the families were proposed to introduce the study product in the regular feeding of their baby up to 2 years’ age. The study started with a screening visit (hospital visit 0, Figure 1). After recording the personal, familial, and medical characteristics, anthropometric data were collected. A cows’ milk symptoms‐based clinical score (CoMiSS) was additionally completed to evaluate the health status of the subject.^16^ Children admitted to the study were evaluated for hematology, creatinine, BUN, albumin, and ferritin by processing patients’ sera from venous blood samples. During week 1, the selected children underwent a DBPCFC including the study product and a corresponding placebo (Neocate, Nutricia, Uthrecht, The Netherlands) in separate days at least 72 hours apart (hospital visits 1 and 2), randomly allocated to food or placebo. The parents of children who passed the challenges were contacted by phone the third day after the last challenge procedure, to investigate any delayed allergic reactions. In case of suspected symptoms of food allergy, a supplemental visit had to be planned. An optional extension phase was designed as an open‐label, single‐arm study (Figure 1). Families of the 16 patients who participated to the optional extension phase (aged to 2 years M 60%), integrated the study product in the infant's diet and were clinically evaluated every 6 months for anthropometric assessments for 1 year. In any case, for these children at the end of the assumption of the study formula, a final visit with the same assessments will be planned. Ethical Committee of the Coordinating Center (Ospedale Pediatrico Bambino Gesù) and each of the local Ethical Committees of the participating centers approved the protocol. It was registered at clinicaltrials.gov under the identifier NCT02414243.

### 2.3 Study procedures

Skin pricking was performed by percutaneous lancing through a drop of fresh milk, and wiped immediately afterward on absorbent paper. Pasteurized, unfrozen milk was obtained locally; 10 mg/mL histamine phosphate in 50% glycosaline and glycosaline on its own (Lofarma, Milano, Italy) were used as positive and negative controls, respectively. A Dome/Hollister‐Stier lancet with a 1‐mm tip was used. Wheal diameters were read through a clear plastic calliper disk scaled in millimeters under ×4 magnification and were interpreted when the wheal margin was included within a complete calliper circle to the nearest millimeter.^17^ A limit of 3 mm was set for SPT positivity. Challenges were performed and interpreted using the EuroPrevall methodology.^18^, ^19^, ^20^ Six incremental doses of food were administered at 20‐minute intervals under clinical supervision. A subjective symptoms‐based clinical score to determine the degree of GI, respiratory, cardiovascular and dermatological reactions, was applied to monitor the acute allergic reactions. The procedure was stopped when clear‐cut objective symptoms were present. The assessment was unblinded after completion of the last challenge day. The children were proposed to use the study product in their regular feeding. Starting from visit 3 (week 2), at each visit, the parents were asked to fill a form of a 3‐day diary with the exact quantities of formula effectively ingested by their infants during the days before the visit. The naked infant was weighted on an electronic Sartorius scale (Sartorius AG, Gottingen, Germany) accurate to ±5 g. Crown‐heel length was measured for children under 2 years on a portable measuring board to the nearest mm. After 2 years, height was measured (in mm) in triplicate at the same time of day (± 4 hours) using a wall‐mounted Holtain Stadiometer. In particular, the longitudinal growth measurements were performed according to a standardized procedure by the same experienced pediatrician. At any time, three measurements were taken for each growth parameter and the average value was considered for the analysis. Weight and length for age percentile: the anthropometric outcomes, such length‐for‐age and weight‐for‐age for subjects up to 2 years old, were given as percentile according to the World Health Organization international growth charts.^21^ A clinician administered the CoMiSS questionnaire to assess the subjective parental perception of patient discomfort. This questionnaire measures five clinical symptoms (crying, regurgitation, stools aspect on Bristol scale, eczema, and urticaria) on a 1 to 6 scale, and respiratory symptoms on a 0 to 3 scale.^16^ CoMiSS symptoms‐based score (SBS) ranges from 0 to 33.

### 2.4 Statistics

The sample size was calculated according to the American Academy of Pediatrics (AAP) guidelines for clinical testing of hypoallergenic formulas. The number of subjects needed to project with 95% confidence (one‐sided interval) that less than 10% of infants will react to the product is 29 subjects if no clinical reactions are observed and 43 subjects if one clinical reaction is observed. These sample size estimates were derived based on binomial distribution techniques using Wald's method for deriving confidence intervals for single proportions (software used: R Version 3.1.0–The R foundation for statistical computing). The analysis on the primary outcome parameter was a per protocol (PP) analysis. For each subject who discontinued the study for any reasons other than allergic reaction to the test formula, an extra subject has been included to meet the adequate number of subjects required for PP analysis. Additional outcomes were obtained on full analysis set (FAS) based on the intention‐to‐treat assumption.

Quantitative parameters have been summarized by descriptive statistics (n, arithmetic mean, standard deviation, minimum, median, and maximum) and qualitative parameters by frequencies and percentages. For n < 3, only arithmetic mean, minimum, and maximum have been displayed. Categorical variables have been presented using nonmissing observations and percentages. Denominators for calculation of percentages have been taken as the number of subjects with nonmissing observations in the specified population unless otherwise stated. Continuous variables have been presented using number of subjects in the analysis population (N), number of subjects with nonmissing observations (n), mean, standard deviation (abbreviated as “SD” in statistical tables), median, minimum, and maximum. For n < 3, only mean, minimum, and maximum have been displayed. The nonparametric randomized analysis of variance (Friedman's test) was used to compare repeated variables (weight and length) during the optional extension phase follow‐up visits at 6 and 12 months. *P* values .05 were considered to indicate statistical significance (two‐tailed tests). The SPSS 14.0 Package for Windows (SPSS Inc, Chicago, IL) was used for the statistical analysis. Unless stated otherwise, statistical tests have been conducted as two‐sided at a level of *P* = .05. *P* values for difference from baseline are calculated using paired *t* test (Table 4).

**Table 4:** Patients comorbidity

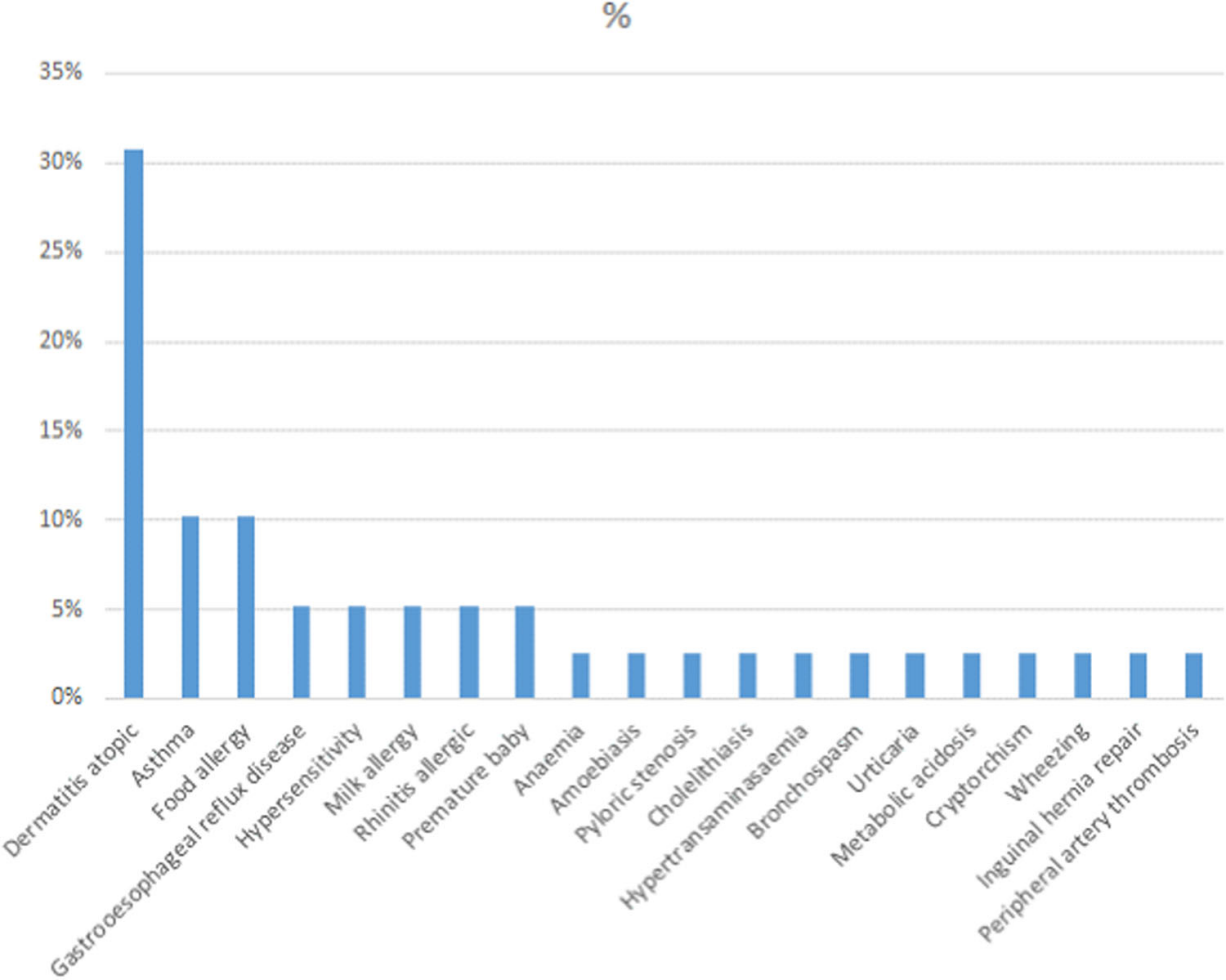

## 3 RESULTS

**Figure 2:**
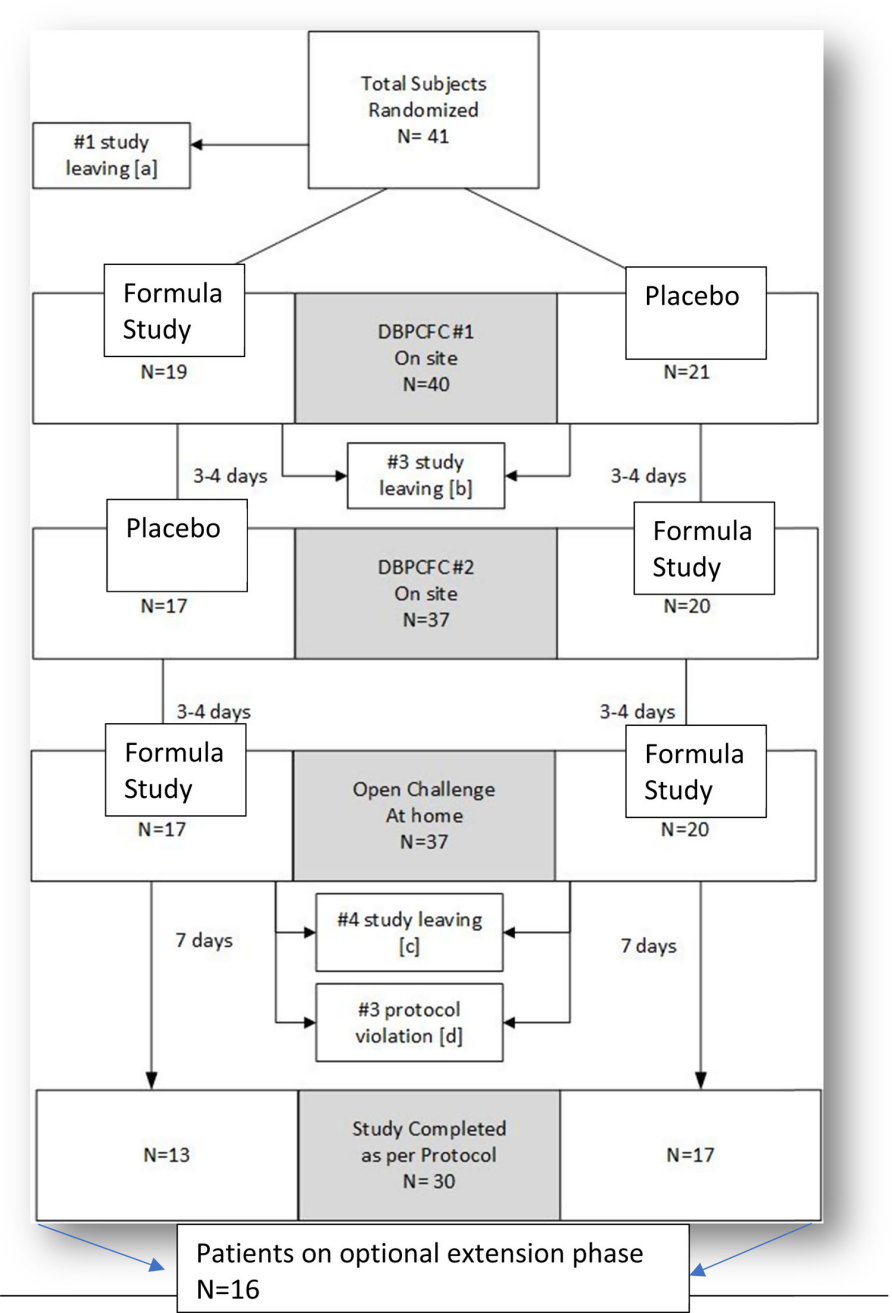
Randomization chart

**Figure 3:**
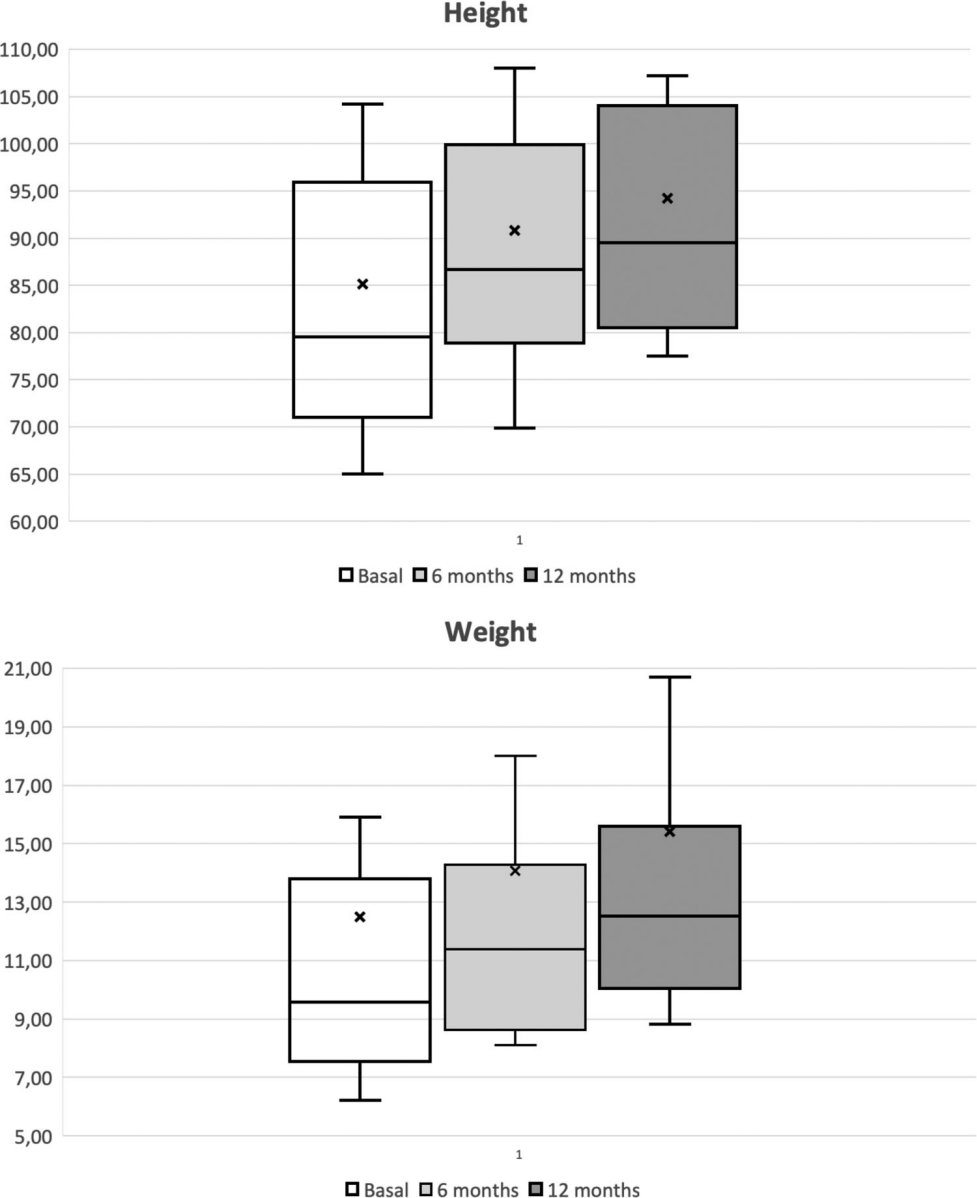
Box and whisker plot showing weight (kg) and height (cm) increase after 6 and 12 months. The bars, box, and whiskers indicate the medians, 25th and 75th centiles and the ranges, respectively

Of 51 children screened between 1 June 2015 and 31 July 2017, 41 children were enrolled and randomized to the formula sequence. Four subjects were not included for screening failure; six were successfully screened, but decided to leave the study before the DBPCFC. As another family decided to leave the study for personal reasons after the randomization during visit 2, 40 children started the DBPCFC phase (FAS population). Three of them refused the formula and discontinued their first DBPCFC procedure before its completion (two in the active group, one on placebo). Thirty‐seven passed the challenge and were admitted to the 7‐day open phase. Four children were excluded before completion of the first week due to insufficient formula intake. Three subjects were not included in the analysis set due a major protocol violation, identified later. In summary, 30 children (17 M and 13 F; median age, 1.58 years; range, 0.08‐12.83 years) completed the open challenge and were able to consume a minimum of 250 mL per day of the study product for at least 7 days (PP population) (see Figure 2 for randomization chart). As no signs or symptoms of allergic reactions were recorded among children assuming either the test or the control formula, the study product met the AAP hypoallergenicity criteria. The lower 95% one‐sided confidence interval for the proportion of subjects who did not experience allergic reactions was CMA‐related symptoms recorded at V1 and V4 by CoMiSS are presented in Table 5. Almost all secondary outcomes in the SBS decreased in a non‐statistically significant way. The total score decreased after the first 2 weeks of treatment by 0.55, without a statistical difference (*P* = .118). A statistically significant difference in weight and height between V4 and the first (after 6 months from V4) and second visit (after 12 months from V4) of the optional extension phase was recorded. The data indicate a significant increase in weight and length (Friedman's test *P* value .00) at 6 and 12 months (see Figure 3). All the hematology and biochemistry parameters were recorded normal for the age at baseline and at visit 4. No significant variations were recorded for hematology values, except MCHC (mean 32.43 ± 1.83 at baseline; 33.36 ± 1.92 after 4 weeks; *P* = .0136) and platelets (397.3 [10^3^/μL] ± 182.5 at baseline; 331.1 ± 158.4 at visit 4; *P* = .0217) they were statistically significant but clinically irrelevant. Immunoglobulins serum levels were within reference ranges. Total and specific IgE were abnormal, according to the inclusion criteria, and did not change over the 2‐week period. All biochemistry data were within reference ranges, without variations over the considered period. There were no serious or nonserious adverse events related to the study treatment or leading to a study treatment discontinuation.

**Table 5:** CoMiSS symptoms summary

Symptom	V1 mean	V4 mean	Change from baseline (*P* value*)
Crying	0.03	0.00	−0.03 (*P* = .325)
Regurgitation	0.13	0.00	−0.14 (*P* = .103)
Stools	0.67	0.41	−0.28 (*P* = .380)
Head, neck, trunk	0.20	0.17	−0.03 (*P* = .662)
Arms, hands, legs, feet	0.17	0.17	0.03 (*P* = .662)
Urticaria	0.03	0.00	−0.03 (*P* = .325)
Respiratory symptoms	0.13	0.03	−0.07 (*P* = .325)
Total score; mean (SD)	1.37 (1.59)	0.75 (0.55)	−0.55 (*P* = .118)
Min‐max	0.00‐6.00	0.00‐4.00	

Abbreviation: CoMiSS, cows’ milk symptoms‐based clinical score.

* Paired *t* test.

## 4 DISCUSSION

The tolerability of AAF is considered high, but occasional allergic reactions have been documented.^22^ This could be due to accidental cross‐contamination with milk proteins when the same equipment is used for producing both AAF and eHF, to the persistence of proteins in starches or fat blend, or to the appearance of neoallergens during manufacturing. In this clinical trial in 30 patients with IgE‐mediated CMA, the study product was able to meet the AAP criteria. No allergic reactions were documented among the 30 subjects that completed the DBPCFC and the open challenge phase. Our results indicate that the producers’ manufacturing practices, a validated cleaning and dragging process to eliminate traces of milk protein from the facility, are sufficient to eliminate potentially dangerous contaminations. We also documented a decreasing trend in CoMiSS, without statistical difference; one can speculate that this is due to the limited period of treatment and observation (2 weeks) and the little population set (the formal sample size was estimated for a different outcome).

Our data document the hypoallergenicity of the study product. The AAP definition of hypoallergenicity applies to eHFs, responsible for reactions among children with CMA.^23^ Not all the AAFs were subjected to formal studies on their hypoallergenicity, but some were. The veteran of these formulae, Neocate (Nutricia) was tested tolerated by 90% of the children with IgE‐mediated CMA (95% confidence) 25 years ago.^24^ Its safety was repeatedly documented among infants with severe food allergies^25^, ^26^, ^27^ and was recently reassessed in a new, textured form.^28^ Similar data were published for the AAF Sineall (Humana, Milan, Italy)^29^ and Novalac (United Pharmaceuticals, Paris, France).^30^, ^31^ All these studies used Neocate as a validated reference product in diagnostic challenges and/or in the treatment phases. Many other AAF formulations are commercially available worldwide, only a minority of which have been studied for hypoallergenicity.^32^

If the efficacy of AAF in CMA management is someway an expected result, greater concerns have been expressed for their nutritional adequacy. Observational studies have shown that infants with CMA show various degrees of growth depression in the first year of life, and the choice of milk substitute may influence their growth rate.^10^ For this reason, great attention has been devoted to the effects of AAFs in terms of growth.^28^, ^33^, ^34^ In that, our data have a limited value. We were able to show a positive difference in weight and height between visit 0 and visit 4, but due to the short periods examined; these results may only suggest that patients’ growth is not hindered by the new formula. The specific effects of test formula on the child's growth need to be assessed in future clinical studies with a different design.

AAF are indicated for the most severe forms of CMA. They are also indicated in case of allergic reaction to eHF. Unlike similar studies^30^, ^31^ we did not select children with severe forms of CMA allergic to eHFs, which is a clear limitation. Another limitation of our study is the lack of a control group, fed an eHF or a different AAF. Yet, these data are important since a number of studies demonstrated that in CMA infants fed AAFs the energy intake may be suboptimal and growth may be impaired compared with healthy children.^35^, ^36^ As the field of application of AAFs may widen with the increasing of the complexity of clinical presentations,^37^ their nutritional adequacy becomes crucial for their positioning. To this end, a longer evaluation of anthropometric data has been performed for patients who joined the study optional phase.

In the majority of clinical conditions of CMA, they are not considered the first‐line treatment due to their cost.^38^ However, beyond the indications from existing guidelines,^39^ AAF are used when symptoms do not fully resolve on eHFs. The majority of clinicians are ready to consider their first‐line use in severe food allergies^40^, ^41^; eosinophilic esophagitis^42^; Food Protein‐Induced Enterocolitis Syndrome^43^; severe eczema^44^; and when infants develop symptoms while breastfeeding.^45^ In conclusion the study product meets the AAP criteria for hypoallergenicity. During the first 2 weeks of its use, gastrointestinal, respiratory, and dermatological symptoms of CMA decreased. It does not reduce the infants’ growth in the short term. The long‐term effects of this new AAF on the child's growth need to be assessed in future clinical studies with a different design, but it is ready to become an available option for children with CMA.
